# Sodium-glucose cotransporter 2 inhibitors antagonize lipotoxicity in human myeloid angiogenic cells and ADP-dependent activation in human platelets: potential relevance to prevention of cardiovascular events

**DOI:** 10.1186/s12933-020-01016-5

**Published:** 2020-04-07

**Authors:** Valentina Spigoni, Federica Fantuzzi, Cecilia Carubbi, Giulia Pozzi, Elena Masselli, Giuliana Gobbi, Anna Solini, Riccardo C. Bonadonna, Alessandra Dei Cas

**Affiliations:** 1 Endocrinology and Metabolism, Department of Medicine and Surgery, University of Parma, Parma, Italy; 2 Department of Medicine and Surgery, Unit of Biomedical, Biotechnological and Translational Sciences (S.Bi.Bi.T.), University of Parma, Parma, Italy; 3 Department of Surgical, Medical, Molecular and Critical Area Pathology, University of Pisa, Pisa, Italy; 4 Division of Endocrinology and Metabolic Diseases, Azienda Ospedaliero-Universitaria of Parma, Parma, Italy; 5 Department of Medicine and Surgery, Division of Endocrinology and Metabolic Diseases, University of Parma and Azienda Ospedaliero-Universitaria of Parma, Via Gramsci 14, 43126 Parma, Italy

**Keywords:** SGLT-2 inhibitors, Myeloid angiogenic cells, Platelets, Atherosclerosis, Sodium-proton exchanger

## Abstract

**Background:**

The clear evidence of cardiovascular benefits in cardiovascular outcome trials of sodium-glucose cotransporter 2 inhibitors (SGLT2i) in type 2 diabetes might suggest an effect on atherosclerotic plaque vulnerability and/or thrombosis, in which myeloid angiogenic cells (MAC) and platelets (PLT) are implicated. We tested the effects of SGLT2i on inflammation and oxidant stress in a model of stearic acid (SA)-induced lipotoxicity in MAC and on PLT activation. The possible involvement of the Na^+^/H^+^ exchanger (NHE) was also explored.

**Method:**

MAC and PLT were isolated from peripheral blood of healthy subjects and incubated with/without SGLT2i [empagliflozin (EMPA) and dapagliflozin (DAPA) 1–100 μM] to assess their effects on SA (100 μM)-induced readouts of inflammation, oxidant stress and apoptosis in MAC and on expression of PLT activation markers by flow-cytometry after ADP-stimulation. Potential NHE involvement was tested with amiloride (aspecific NHE inhibitor) or cariporide (NHE1 inhibitor). Differences among culture conditions were identified using one-way ANOVA or Friedman test.

**Results:**

NHE isoforms (1,5–9), but not SGLT2 expression, were expressed in MAC and PLT. EMPA and DAPA (100 μM) significantly reduced SA-induced inflammation (*IL1β*, *TNFα*, *MCP1*), oxidant stress (*SOD2*, *TXN*, *HO1*), but not apoptosis in MAC. EMPA and DAPA (both 1 μM) reduced PLT activation (CD62p and PAC1 expression). SGLT2i effects were mimicked by amiloride, and only partially by cariporide, in MAC, and by both inhibitors in PLT.

**Conclusions:**

EMPA and DAPA ameliorated lipotoxic damage in stearate-treated MAC, and reduced ADP-stimulated PLT activation, potentially via NHE-inhibition, thereby pointing to plaque stabilization and/or thrombosis inhibition as potential mechanism(s) involved in SGLT2i-mediated cardiovascular protection.

## Background

Sodium-glucose cotransporter 2 inhibitors (SGLT2i) act by inhibiting the Na/glucose co-transporter 2 (SGLT2) in the kidney proximal tubule leading to glycosuria with consequent improvement in glucose control, weight reduction and decrease in blood pressure.

Cardiovascular outcome trials (CVOT) [1–3], conducted at almost glucose equipoise in patients with type 2 diabetes mellitus (T2D), have shown that, in patients with established atherosclerotic cardiovascular disease (ASCVD), but not in patients with multiple risk factors (MRF), SGLT2i as a whole reduce the risk of major adverse cardiovascular events (MACE), suggesting a class effect [4]. Beneficial effects were also exerted on hospitalization for heart failure and worsening of chronic kidney disease (CKD), with no apparent distinction between patients with established ASCVD or with MRF only [4]. These results were confirmed and expanded in the CREDENCE trial, conducted in patients with type 2 diabetes and albuminuric chronic kidney disease [5]. Furthermore, dapagliflozin was able to reduce the rates of cardiovascular death and hospitalization for heart failure also in nondiabetic patients with low ejection fraction heart failure [6].

The mechanism(s) responsible for these clinical benefits might be not fully explained with pathogenetic scenarios based on the canonical SGLT2i effects [7]. According to recently reported evidence in cardiomyocytes, SGLT2i can inhibit Na^+^/H^+^ exchanger (NHE), resulting into a fall in intracellular Na^+^ and Ca^2+^ while increasing mitochondrial Ca^2+^ concentrations, ultimately optimizing cardiac mitochondrial function and energetics [8]. This off-target effect of SGLT2i may explain, at least in part, the beneficial influence of SGLT2i treatment on heart failure [4, 6]. On-target effects of SGLT2i, which reduce the burden of the hyperfiltrating glomerulus, and/or off-target effects again through NHE inhibition in renal tubular cells may help to explain the benefits exerted by SGLT2i on CKD [4, 5]. However, none of these lines of evidence can account for the reduction in MACE brought about by SGLT2i in patients with established ASCVD [4, 5].

The relatively early separation of the curves of MACE (within about 6 and 12 months in the EMPAREG-OUTCOME [1] and CANVAS [9] trials, respectively), between patients on placebo and patients on SGLT2i, and the general belief that MACE are due primarily to disruption/complication of vulnerable atherosclerotic plaques led us to hypothesize that SGLT2i could modify the biology of cells involved in the atherosclerotic plaque behavior. Two such cell types are myeloid angiogenic cells (MAC) and platelets (PLT). MAC, most commonly named endothelial progenitor cells, are involved in atherogenesis/atherosclerosis under many respects: (1) circulating MAC number is inversely associated to and predicts cardiovascular events [10–12]; (2) infusion of MAC in rodents mitigates the atherosclerotic burden due to hypercholesterolemia [13]; (3) in contrast, immature MAC are overrepresented in the microvasculature of vulnerable plaques in which they may play a detrimental role [14]. Thus, both bioavailability and functional status of MAC are important to define their role in atherosclerosis.

Lipotoxicity, often triggered by chronically elevated free fatty acid (FFA) levels, is a well-known mechanism underlying the association among IR (and T2D), endothelial dysfunction and increased CV risk [15]. We recently published [16] an experimental model of MAC lipotoxicity ex vivo in which physiological concentrations of stearic acid (SA) induced a pro-inflammatory response, oxidant stress and (lipo)apoptosis through the activation of endoplasmic reticulum stress signaling pathways, ultimately leading to impaired endothelial repair processes.

The roles played by PLT in atherogenesis [17] and atherothrombosis [18] are well known. Specifically, PLT recruitment, activation and aggregation at vulnerable plaques are considered key events leading to clot formation and eventually resulting in acute cardiovascular and cerebrovascular ischemic events [19].

In this study we tested the hypotheses that: (1) the beneficial effects of SGLT2i on CV outcomes might be also mediated by some direct action on MAC and/or PLT activation; (2) the inhibition of NHE might represent one of the possible mechanisms underlying these putative effects.

## Methods

In this in vitro study we tested the effects of SGLT2i on inflammation and oxidant stress in a model of stearic acid induced lipotoxicity in MAC and on PLT activation. The possible involvement of NHE was also explored.

### Myeloid angiogenic cell (MAC) isolation and culture conditions

#### MAC isolation

Myeloid angiogenic cells (MAC) were isolated and cultured according to published methods [11, 20] as previously described [16, 21–23]. Briefly, peripheral blood mononuclear cells (PBMCs) from healthy donors’ buffy-coats were isolated by Lymphoprep (Euroclone, Milano, Italy) density gradient centrifugation and cultured into six-well tissue fibronectin-coated plates at a density of 10^7^ cells/well. PBMCs were grown in endothelial cell growth medium-2 with supplements (EGM-2 bullet kit, Lonza, Milano, Italy) at 37 °C in a humidified 5% CO_2_ incubator for 7 days. On day 7, MAC (CD45^+^/CD14^+^/CD64^+^/CD31^+^/KDR^+^/CD34^−^ phenotype) appeared in culture as adherent cells displaying an elongated spindle-shaped morphology.

#### Culture conditions

At day 7, MAC were pre-treated with/without empagliflozin (EMPA) or dapagliflozin (DAPA) (both from 1 to 100 μM) for 16 h in the presence/absence of 100 µM of stearic acid (SA), as reported in our previous paper [16]. Vehicle (DMSO 0.1%)-treated cells were used as controls. Where appropriate, 4 mM *N*-acetyl-cysteine (NAC)—an anti-oxidant molecule [24]—was added in culture. In experiments aimed at studying the role of NHE, the unselective NHE inhibitor (NHEi) amiloride (100 µM) or the specific NHE-1 blocker cariporide (10 µM) were used. All the compounds were dissolved in DMSO, except for NAC which was dissolved in H_2_O.

#### Stearate solution preparation

Stearate stock solution was prepared by dissolving SA (Sigma-Aldrich, St Louis, MO, USA) in NaOH 0.1 M at 73 °C for 30 min. SA was subsequently complexed to 10% (wt/vol) bovine serum albumin (BSA) (FFA:BSA molar ratio = 3.3:1) in order to obtain 5 mM working solution as previously described [16, 25].

### Platelet (PLT) purification

PLT were purified from healthy donors’ peripheral blood by immunomagnetic negative selection with anti-CD45 microbeads to deplete nucleated cells and used for mRNA and proteins extraction, as previously described [26]. Briefly, blood samples were centrifuged at 160*g* for 20 min to obtain platelet rich plasma which was stained with the magnetic beads-coated mAb anti-CD45 for 20 min at RT on a rotator. Samples were placed in a magnetic field and PLT were collected as negative fraction. Purified PLT were counted and processed for RNA and protein extraction.

### Viability assay in MAC

To evaluate potential SGLT2i cytotoxic action in MAC, we assessed cell viability by using VisionBlue fluorescence cell viability assay kit (Biovision, Mountain View, CA), following manufacturer’s instructions, as already reported [16, 21, 23]. Briefly, MACs were cultured in 96-well culture plates at a density of 2.5 × 10^5^ cells per well (in a volume of 100 µl of culture medium) and exposed to EMPA and DAPA (1, 10 and 100 µM) for 16 h. Ten microliters of VisionBlue reagent was added in each well and, following incubation (2 h at 37 °C), the fluorescent product was measured (excitation: 540 nm, emission: 586 nm) using Cary Eclipse spectrophotometer (Varian/Agilent, Santa Clara, CA, USA). Data were normalized for control values.

### Pro-inflammatory and oxidant stress marker gene expression in MAC

To study the possible anti-inflammatory action of SGLT2i in SA-treated MAC, pro-inflammatory marker gene expression was assessed by quantitative PCR (qPCR). Cells were lysed with QIAzol lysis reagent and total RNA was extracted (miRNeasy Mini Kit Qiagen Ltd, West Sussex, UK) after 3 h—and 6 h only for SGLT2i experiments—of SA-exposure, time point(s) at which SA noticeably increases inflammation [16]. cDNA was obtained by iScript Reverse Transcription Kit (Bio-Rad Laboratories, Inc. Hercules, Ca, USA) starting from 250 ng of total RNA. Interleukin *(IL)*-*1β, IL*-*8*, tumor necrosis factor-α (*TNF*-*α*), and monocyte chemoattractant protein-1 (*MCP*-*1*) gene expression was tested using SsoAdvanced Universal Probes Supermix (Bio-Rad) with TaqMan primers and probes (IL-1β: Hs01555410_m1; IL-8: Hs00174103_m1; TNF-α: Hs00174128_m1; MCP-1: Hs00234140_m1) (Applied Biosystems, Carlsbad, CA, USA) on a CFX Connect Real-Time (Bio-Rad), as previously reported [16, 23]. Specific thermal cycling conditions were used: 98 °C for 30 s, followed by 40 amplification cycles (95 °C for 3 s; 60 °C for 20 s). Gene expression values were calculated based on the ΔΔCt method and *GAPDH* (Hs03929097_g1) and/or *RPS18* (Hs01375212_g1) were used as reference gene (both the housekeeping genes achieved high expression stability criterion with M value < 0.5 [27]). At least four independent experiments were performed and samples were analyzed in triplicate.

The same procedures were used to assess superoxide dismutase (*SOD*-*2*: Hs00167309_m1), thioredoxin (*TXN*: Hs00828652_m1) and heme oxygenase (*HO*-*1:* Hs01110250_m1) gene expression as a measure of oxidant stress in EMPA/DAPA or NAC pre-treated MAC followed by 6 h of SA-stimulation.

### Pro-inflammatory molecule secretion in MAC

IL-8, MCP-1 and TNF-α concentrations in cell culture supernatants were quantified by a magnetic bead multiparameter kit (R&D Systems, Minneapolis, MN, USA) according to kit instructions and analyzed on a MagPix instrument (Luminex Corporation), as previously reported [16].

IL-1β quantification was assessed by IL-1 beta High Sensitivity ELISA Kit (Thermo Fisher Scientific, Waltham, MA U.S.A.) following manufacturer’s instruction. The reported limit of detection was 0.05 pg/ml and intra- and inter-assay coefficients of variation were 6.7% and 8.1%, respectively. IL-1β concentration in cell supernatants was calculated by using a standard curve and by measuring the absorbance at 450 nm in a microplate reader (Multiskan™ FC Microplate Photometer, Thermo Scientific). At least four independent experiments were performed and samples were analyzed in duplicate.

### Apoptosis assessment in MAC

Effects of the SGLT2i in preventing lipoapoptosis were assessed by using Caspase-Glo 3/7 assay, according to manufacturer’s instructions (Promega Corporation, Madison, WI, USA). MAC were cultured in 96-well culture plates (2.5 × 10^5^ cells/well) and pre-treated 16 h with/without EMPA or DAPA in presence/absence of SA 100 µM. After 24 h, cells were incubated with 100 μl of Caspase-Glo 3/7 reagent at 37 °C for 30 min and luminescence was measured by Cary Eclipse fluorescence spectrophotometer (Varian/Agilent, Santa Clara, CA, USA). Fold increase in caspase activity was normalized to the activity obtained from control. Three independent experiments were performed and samples were analyzed in triplicate.

### Platelet activation assay

PLT activation tests were performed on whole blood keeping samples at room temperature until processing (< 4 h from venipuncture) and during incubation steps. Reagents were added sequentially to the samples avoiding washing and centrifugation in order to minimize artifactual in vitro activation, as recommended [28]. Activation was assessed by flow cytometry and PLT activation markers, CD62p and PAC-1, were analyzed as previously described [29]. Briefly, 5 µL of whole blood was diluted 1:100 in PBS and incubated with EMPA (1 µM) or DAPA (1 µM) or Amiloride (100 µM) or Cariporide (10 µM) or DMSO (as control), for 15 min a room temperature (RT). PLT were then stimulated with/without ADP 5 µM and monoclonal antibodies anti p-Selectin (FITC anti-human CD62p, BD Biosciences, Franklin Lakes, NJ, USA) or directed to the activated form of αIIbβ3 complex (FITC anti-human PAC1, BD Biosciences) were added. After 30 min of incubation at room temperature in the dark, samples were fixed by 2% paraformaldehyde. Analysis was performed by an Epics XL flow cytometer (Beckman Coulter, Fullerton, CA, USA) and the Expo ADC software (Beckman Coulter).

### SGLT2 expression in MAC e PLT

To assess whether the observed effects in MAC and PLT were mediated by SGLT2, gene expression in MAC and protein quantification in PLT were performed.

#### Digital PCR in MAC

The absolute quantification of *SGLT1* and *SGLT2* gene expression in MAC was performed by digital PCR QX200 (Bio-Rad, Hercules, California USA). Three hundred ng of total RNA was reverse-transcribed using High Capacity cDNA Reverse Transcription Kit (Applied Biosystems) and cDNA was amplified with TaqMan Gene Expression assay (SGLT1:Hs01573790_m1 and SGLT2: Hs00894642_m1—Life Technologies Italia) following the manufacturer’s protocol. A total of 20.000 droplets were approximately generated for each sample. A conventional thermal cycler was used for the next PCR step using the following thermal cycling conditions: 1 cycle at 95 °C for 10 min, 44 cycles at 95 °C for 30 s and 60 °C for 1 min, 1 cycle at 98 °C for 10 min, all at a ramp rate of 2 °C/s. The number of target molecules in each sample was estimated according to Poisson distribution using the Bio-Rad QuantaSoft software. The expression of the target genes was normalized to *GAPDH* reference gene.

#### SGLT2 protein expression in PLT

Total proteins were extracted from purified PLT. Briefly, 4.5 × 10^5^ PLT were suspended in a cell lysis buffer supplemented with fresh protease/phosphatase inhibitors and protein concentration was determined. Proteins from PLT and HEK293, as positive control, were separated in 5% SDS-acrylamide gels and blotted onto nitrocellulose membranes. Membranes were blocked and incubated overnight at 4 °C with 1:1000 diluted rabbit polyclonal anti-SGLT2 (Cell Signaling Technologies, Danvers, MA, USA). Mouse mAb anti-GAPDH (1:5000 dilution—Millipore, Burlington, MA, USA) was used as loading control. Membranes were then incubated with 1:2000 peroxidase-conjugated anti-mouse (Sigma Aldrich) secondary antibody and detected by chemiluminescence using ECL Supersignal West Pico Chemiluminescent Substrate detection system (Thermo Scientific, Waltham, MA, USA). NIH ImageJ software was used to scan images and quantify protein expressions.

### NHE gene expression analysis in MAC and PLT

Due to the lack of SGLT2 expression in both MAC and PLT we hypothesized the potential involvement of NHE—as a SGLT2-independent effect—based on recent data from literature [8].

NHE isoform (*NHE*-*1*, *NHE*-*2*, *NHE*-*3*, *NHE*-*4*, *NHE*-*5*, *NHE*-*6*, *NHE*-*7*, *NHE*-*8*, *NHE*-*9*) gene expression was assessed by qPCR in both untreated MAC (for detailed methodology see “pro-inflammatory and oxidant stress marker gene expression in MAC”) and PLT.

Purified PLT were treated with an appropriate amount of TRIzolTM (Life Technologies, Carlsbad, CA, USA) for cell lysis and RNA extraction, following manufacturer’s instructions. Briefly, chloroform was added to TRIzolTM-treated samples and centrifuged at 12,000*g* for 15 min at 4 °C. The aqueous phase, containing RNA, was added with an equal volume of isopropanol. After incubation, the samples were centrifuged at 12,000*g* for 15 min at 4 °C, to obtain RNA pellets. The isolated RNA was reverse transcribed by High capacity RNA-to-cDNA kit (Applied Biosystems) and then tested for gene expression (TaqMan system, Applied Biosystems) using *ACTB* (Applied Biosystems) as a reference gene.

### NHE protein expression analysis in MAC and PLT

The expression of the NHE isoforms hitherto detected by qPCR—NHE-1, NHE-5, NHE-6, NHE-7, NHE-8, NHE-9—was confirmed by western blot in both untreated MAC and PLT.

A total of 50–100 µg of proteins from PLT and MAC were separated in Any kD MiniProtean TGX gels (Bio-Rad) and blotted onto PVDF membranes. After a blocking step, primary antibodies diluted 1:500 for NHE 5, 6, 8, 9 (Cohesion Biosciences, London, UK), NHE 1, 7 (Abcam, Cambridge, UK) and GAPDH (Millipore) were incubated overnight at 4 °C. The following day, membranes were reacted with species-specific secondary antibodies HRP-conjugated (Millipore, diluted 1:5000); detection was performed using Clarity Western ECL and Chemidoc Imaging System (Bio-Rad). For each different antibody, a positive control (cell extract of mouse brain, kidney and liver or human lymphocytes) was used, as suggested in the datasheets. Due to technical limitation, PLT protein extract and positive controls cannot be shown simultaneously.

### Statistical analysis

Data are presented as mean ± SEM and differences were identified using one-way ANOVA (followed by Tukey’s post hoc contrasts). Pro-inflammatory protein secretion data, which are skewed distributed, are presented as median (min–max) and differences analyzed by Friedman followed by Dunn’s multiple comparison test. Statistical significance was set at p < 0.05 (two-sided). Data analysis was performed using SPSS version 25 (SPSS Inc/IBM, Chicago, Ill, USA).

## Results

A diagram with main results and hypothesized mechanisms has been added in Additional file 1: Figure S1.

### Effects of SGLT2i on SA-induced inflammation, oxidant stress and apoptosis in MAC

Pilot experiments excluded any cytotoxic effect of SGLT2i (from 1 to 100 µM) in MAC (data not shown).

**Fig. 1:**
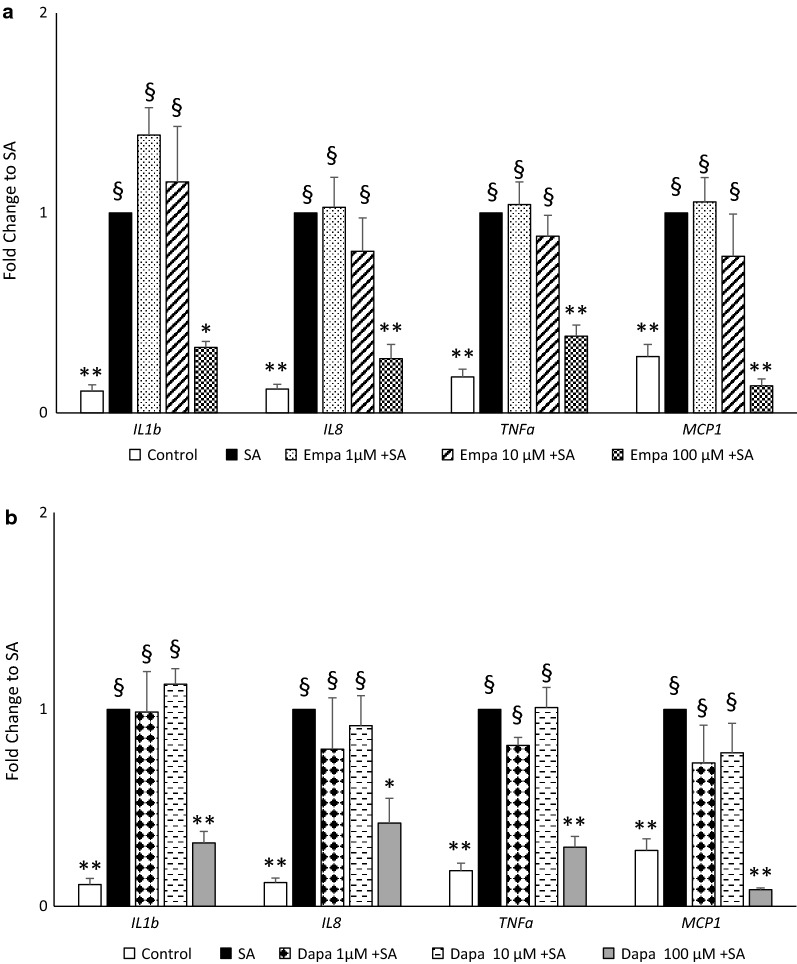
Pro-inflammatory cytokine/chemokine gene expression. Gene expression of pro-inflammatory cytokine/chemokine was assessed in MAC pre-treated with empagliflozin (**a**) or dapagliflozin (**b**) at different concentrations (1–10–100 µM) for 16 h followed by 3 h of stearate 100 µM incubation (*Empa* empagliflozin, *Dapa* dapagliflozin, *IL* interleukin, *TNFa* tumor necrosis factor-α, *MCP* monocyte chemoattractant protein, *SA* stearate). Data are expressed as mean ± SEM from at least 4 independent experiments (*p < 0.05, **p < 0.01 vs SA; ^§^p < 0.01 vs control)

**Fig. 2:**
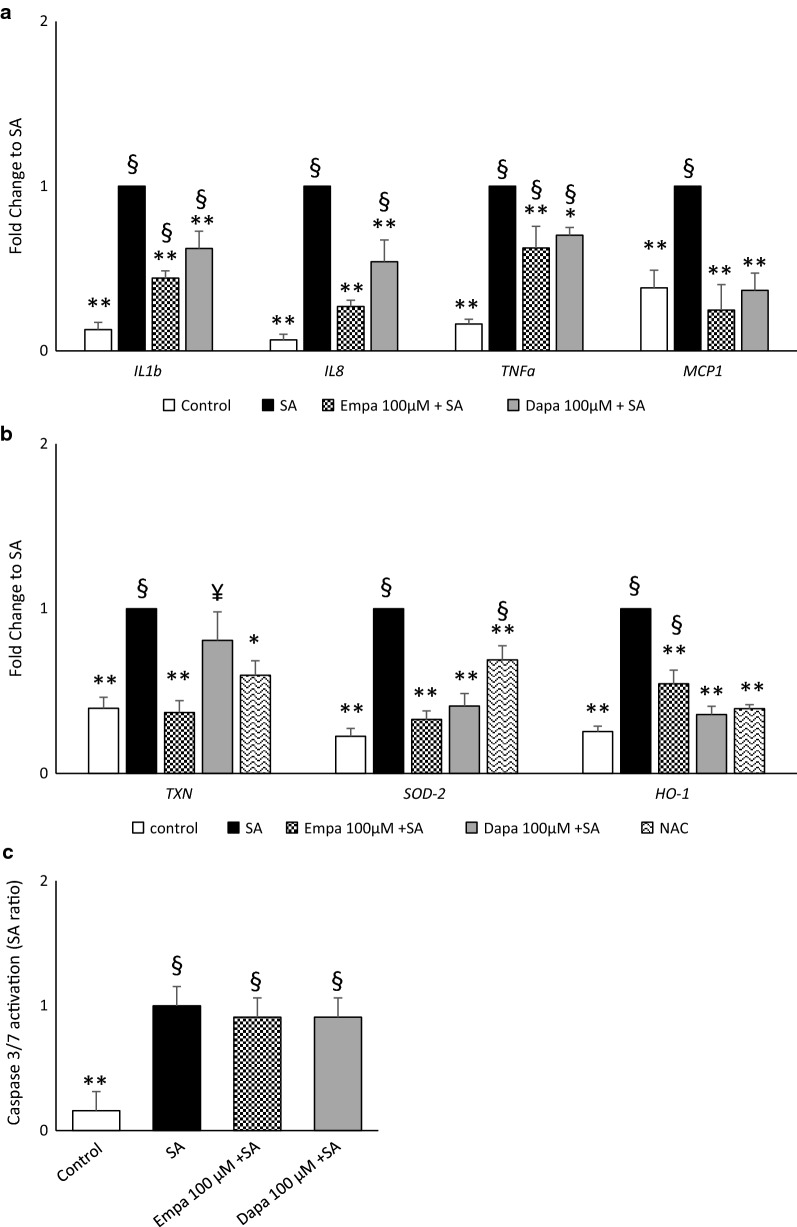
Effects of SGLT2i on inflammation, oxidative stress marker and apoptosis in SA-treated MAC. Gene expression of pro-inflammatory cytokine/chemokines (**a**) and oxidative stress markers (**b**) was assessed in MAC pre-treated with empagliflozin (Empa) or dapagliflozin (Dapa) 100 µM for 16 h—or *N*-acetyl-cysteine (NAC 4 mM) for 1 h—followed by 6 h of stearate (SA) 100 µM incubation (*IL* interleukin, *TNFa* tumor necrosis factor-α, *MCP* monocyte chemoattractant protein, *TXN* thierodoxin, *SOD* superoxide dismutase, HO heme oxygenase). Caspase 3/7 activation was assessed in MAC pre-treated with empagliflozin or dapagliflozin for 16 h followed by 24 h of SA incubation (**c**). Data are expressed as mean ± SEM of at least 3 independent experiments (*p < 0.05, **p < 0.01 vs SA; ^¥^p < 0.05, ^§^p < 0.01 vs control)

Pre-incubation (for 16 h) with EMPA or DAPA significantly depressed SA-induced pro-inflammatory marker (IL-1β, IL-8, TNF-α and MCP-1) gene expression at 3 h (Fig. 1a and b, respectively). This reduction in inflammation, by both EMPA and DAPA, was observed only at the highest concentration tested (100 µM) with cytokine/chemokine gene expression almost dropping to baseline values. Based on these results, all the subsequent experiments in MAC were carried out at SGLT2i concentration of 100 µM. The reduction of SA-stimulated pro-inflammatory marker gene expression by EMPA and DAPA was confirmed also after 6 h of SA incubation (Fig. 2a). We then tested the potential role of SGLT2i in curbing SA-induced oxidant stress. As shown in Fig. 2b, both EMPA and DAPA significantly reduced SA-stimulated gene expression of *SOD*-*2* and *HO*-1, while *TXN* expression was significantly lowered only by EMPA. The inhibition of oxidant stress by SGLT2i was comparable to, or even greater than, that of the anti-oxidant NAC (4 mM).

As SGLT2i curbed inflammation and oxidant stress due to SA-dependent lipotoxicity, we assessed whether EMPA and DAPA might also lower SA-associated apoptosis (caspase 3/7 activation). However, neither DAPA nor EMPA could restrain SA-triggered apoptosis (Fig. 2c).

### Effects of SGLT2i on SA-induced pro-inflammatory molecule secretion

**Fig. 3:**
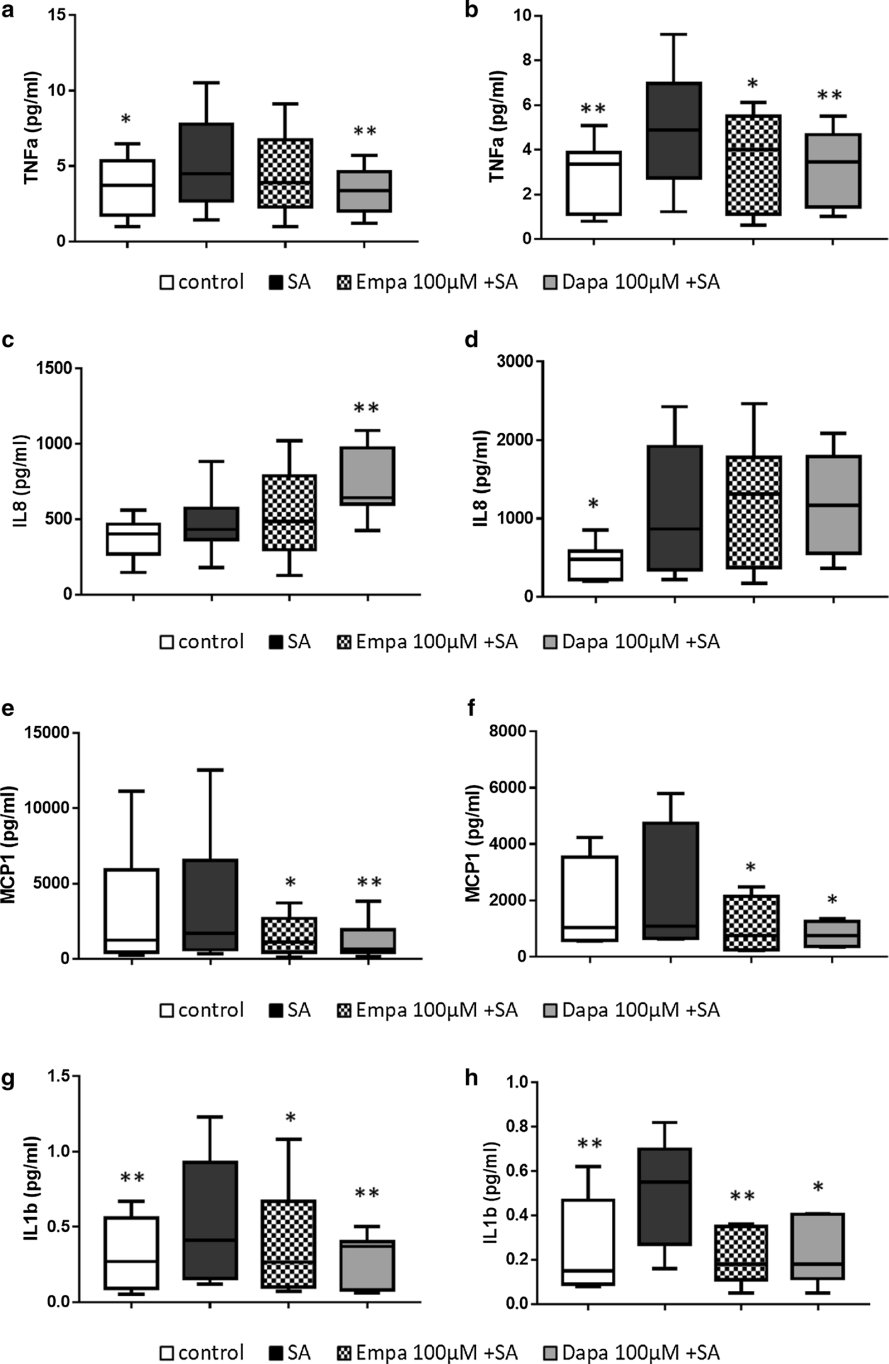
Effects of SGLT2i on pro-inflammatory cytokine/chemokine secretion in SA-treated MAC. Protein expression levels of TNF-α (**a** and **b**), IL-8 (**c** and **d**), MCP1 (**e** and **f**) and IL-1β (**g** and **h**) were assessed in MAC pre-treated with empagliflozin (Empa) or dapagliflozin (Dapa) 100 µM for 16 h followed by 3 h (**a**, **c**, **e**, **g** on the left) or 6 h (**b**, **d**, **f**, **h** on the right) of stearate (SA) 100 µM incubation (*IL* interleukin, *TNFa* tumor necrosis factor-α, *MCP* monocyte chemoattractant protein). Bars in the boxes represent the median (min to max values) of at least 4 independent experiments (*p < 0.05, **p < 0.01 vs SA)

We then assessed pro-inflammatory molecule (IL-1β, IL-8, TNF-α and MCP-1) secretion in the supernatants of MAC pre-incubated for 16 h with SGLT2i followed by SA (100 µM) stimulation for 3 h and 6 h (Fig. 3 panels on the left and on the right, respectively). SGLT2i curbed SA-induced TNF-α and IL-1β secretion (except for EMPA which did not reduce TNF-α production at 3 h of SA-incubation), showing a correspondence between gene expression and protein secretion data. SGLT2i strongly reduced also MCP-1 levels, although SA did not trigger its release, neither at 3 h nor at 6 h. Of note, SA-induced IL-8 secretion was not reduced by SGLT2i, rather the presence of DAPA further increased IL-8 release in culture medium at 3 h of SA-incubation (Fig. 3c).

### Inhibitory effect of SGLT2i on platelet activation

**Fig. 4:**
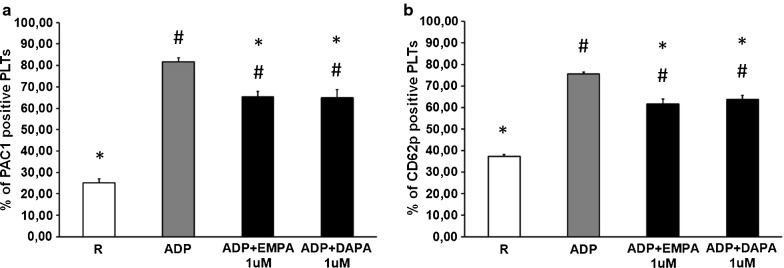
Flow-cytometry analysis of SGLT2i effects on platelet activation. Surface expressions of αIIbβ3 complex, recognized by PAC1 binding (**a**), and CD62p (**b**) were tested on resting platelets (R) and platelets activated with 5 µM ADP, alone (ADP) or in combination with Empagliflozin 1uM (ADP+ EMPA 1uM) or with Dapagliflozin 1uM (ADP+ DAPA 1uM), by flow cytometry. Data are expressed as mean of positive platelets (%) ± SEM from at least 3 independent experiments. ^#^p < 0.05 vs R; *p < 0.05 vs ADP

We tested SGLT2i effects on PLT activation state by analyzing the expression of two PLT receptors: CD62p, a component of the platelet α-granule membrane expressed on activated PLT surface only after degranulation, and αIIbβ3 complex, a fibrinogen receptor, which undergoes a conformational change in response to PLT activation, selectively recognized by the monoclonal antibody PAC-1. As expected, ADP stimulation strongly increased PAC1 (Fig. 4a) and CD62p (Fig. 4b) expression on PLT. Of note, the addition of EMPA and DAPA significantly reduced expression of both CD62p and αIIbβ3 on PLT surface, as compared to ADP alone. These data show that EMPA and DAPA inhibited PLT response to in vitro pro-thrombotic stimulation.

### SGLT2 and NHE expression in MAC and PLT

Digital PCR, qPCR and western blot data showed null expression of SGLT2 both in MAC and in PLT (data not shown). Furthermore, almost absent SGLT1 expression was detected in MAC by digital PCR.

We therefore tested the hypothesis that SGLT2i could act in MAC and/or PLT through NHE inhibition. We first assessed NHE isoform (from 1 to 9) [30] gene expression by qPCR in MAC and PLT and we found a similar pattern of expression of NHE isoforms in MAC and PLT: both cell populations express NHE1, 8 and 9 and are negative for NHE2, 3, 4 isoforms. On the contrary NHE6 and 7 isoforms are express only by MAC, while NHE5 is express only by PLT.

**Fig. 5:**
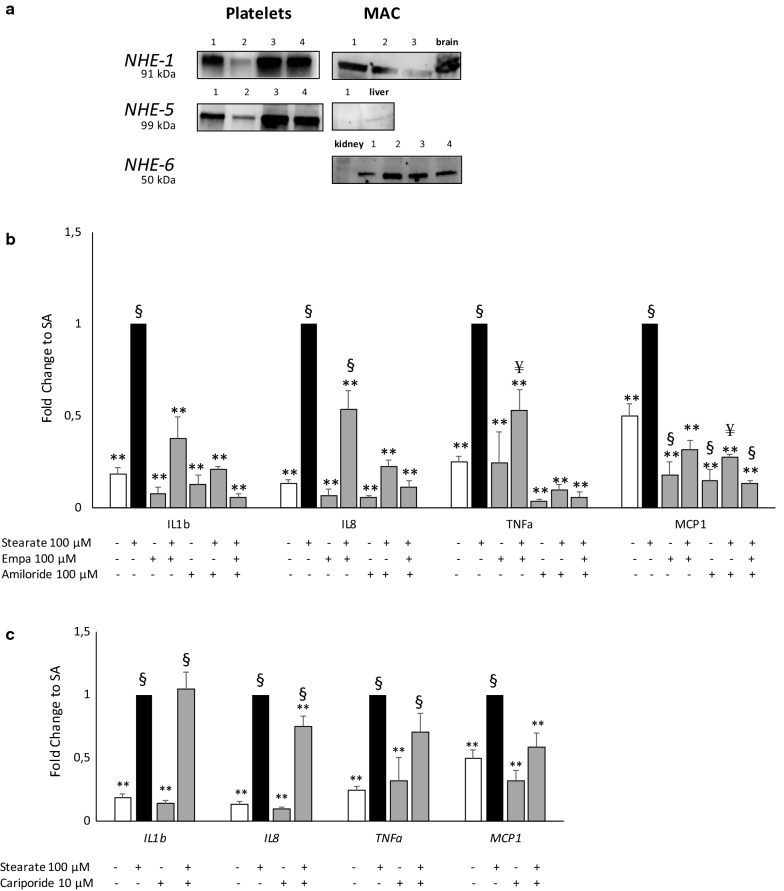
NHE expression and effect of NHE inhibition on SA-induced inflammation in MAC. NHE 1, 5, 6 expression from total protein extracts of PLT and MAC was assessed by western blotting (**a**). For each different antibody a positive control (cell extract of mouse brain, kidney and liver) was used, as indicated in the datasheets. Due to technical limitation in PLT protein extract the positive control cannot be shown, simultaneously. Gene expression of pro-inflammatory cytokine/chemokine was assessed in MAC pre-treated with empagliflozin and/or amiloride both 100 µM (**b**) or cariporide 10 µM (**c**) for 16 h followed by 3 h of stearate 100 µM incubation. (*Empa* empagliflozin; *IL* interleukin, *TNFα* tumor necrosis factor-α, *MCP* monocyte chemoattractant protein, *SA* stearate). Gene expression data are expressed as mean ± SEM from at least 3 independent experiments (*p < 0.05, **p < 0.01 vs SA; ^¥^p < 0.05 ^§^p < 0.01 vs control)

Western blots confirmed NHE1 expression in both cell types, whereas NHE 5 expression was present only in PLT, and NHE 6 only in MAC (Fig. 5a), accordingly to gene expression results. Data on NHE isoform 7, 8 and 9 resulted partially elusive: just one, out of four, MAC protein extract was positive for NHE 7, and NHE 8 and 9 were not detectable in all the tested samples in both MAC and PLT (Additional file 2: Figure S2).

### The NHE inhibitor amiloride, but not cariporide, reduced SA-stimulated inflammation in MAC

To explore the hypothesis that NHE might be a potential transducer of SGLT2i anti-lipotoxic action in MAC, we tested the effect of amiloride, a wide spectrum inhibitor of Na^+^/H^+^-exchangers, on SA-dependent inflammation, in the absence/presence of EMPA.

Amiloride apparently mimicked the blunting effects of EMPA on SA-stimulated expression of IL-1β, IL-8, TNF-α and MCP-1 (Fig. 5b), suggesting a common mechanism of action, i.e. NHE inhibition. It should be noted, however, that EMPA plus amiloride showed an additive effect in curbing SA-induced inflammation.

We then replicated these experiments with cariporide, a selective inhibitor of NHE 1, which is the isoform present in most of the cells and is thought to be responsible for maintaining cellular pH and volume [30]. As shown in Fig. 5c, the presence of cariporide moderately decreased SA-induced *IL*-*8* and *MCP*-*1* expression (but not that of *IL*-*1β* and *TNF*-*α*), thereby suggesting that inhibition of NHE-1 by SGLT2i in the best case could account for only part of anti-inflammatory and anti-oxidant action of SGLT2i in MAC exposed to the lipotoxic effects of SA.

### Amiloride and cariporide reduced ADP-stimulated platelet activation

**Fig. 6:**
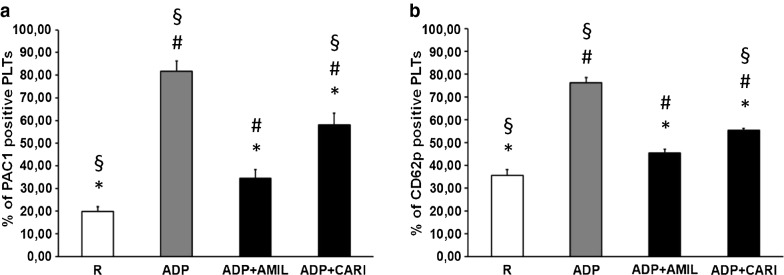
Effects of NHE inhibition on platelet activation. Surface expressions of αIIbβ3 complex, recognized by PAC1 binding (**a**), and CD62p (**b**) were assessed on resting platelets (R) and platelets activated with 5 µM ADP, alone (ADP) or in combination with Amiloride 100 µM (ADP + AMIL) or with Cariporide 10 µM (ADP + CARI), by flow cytometry. Data are expressed as mean of positive platelets (%) ± SEM from at least 3 independent experiments. ^#^p < 0.05 vs R; *p < 0.05 vs ADP; ^§^p < 0.05 vs ADP + AMIL

NHE isoforms contribute to PLT activation and their inhibition affects PLT degranulation and formation of platelet–leukocyte aggregates [31]. Accordingly, in vitro treatment with amiloride and, more specifically, with cariporide (specific NHE1 inhibitor) reduced PLT response to ADP-stimulation (Fig. 6a and b), mimicking the effects of EMPA and DAPA (Fig. 4a and b). Of note, the expression of CD62p and PAC1 in amiloride-treated PLT was similar to that of unstimulated PLT and significantly lower than EMPA, DAPA and cariporide in ADP-stimulated PLT, suggesting stronger PLT inhibition by amiloride than any other substance tested in this study.

## Discussion

In spite of the striking evidence of SGLT2i-mediated cardioprotection in clinical trials [1–3, 5, 6], the underlying mechanisms are still unclear. To the best of our knowledge, no published human studies have explored the effects of SGLT2i on the function(s) of cell types directly involved in plaque (in)stability/thrombosis.

Our study demonstrates that two SGLT2 inhibitors, empagliflozin and dapagliflozin, can curb in vitro (a) inflammation and oxidant stress induced by stearic acid exposure in myeloid angiogenic cells and (b) ADP-dependent platelet activation. These hitherto undescribed effects are SGLT2 independent and may involve one or more isoforms of NHE, as possible molecular transducer(s) of EMPA and DAPA action in both cell populations.

### Clinical relevance of SGLT2 inhibition in MAC and PLT

The SGLT2i-mediated reduction of lipotoxicity in MAC may be particularly relevant in patients with type 2 diabetes, in which lipotoxicity is a key pathogenetic mechanism, with regard to the prominent role played by these cells in maintaining endothelial homeostasis. Furthermore, apart from the predictive negative prognostic power of the number of circulating MAC with regards to CV events and mortality [32, 33] dysfunctional MAC are believed to orchestrate neovascularization of the atherosclerotic plaque, thereby enhancing its risk of rupture and thrombosis [14]. Similarly, and perhaps synergistically, EMPA and DAPA, by opposing ADP-activation of PLT, may reduce the risk of atherothrombosis in the patient with type 2 diabetes, who is well known to harbor overactive platelets with enhanced sensitivity to agents inducing PLT aggregation [34].

The mutual interaction and intensification of inflammation and thrombosis has been described in atherosclerotic processes [35, 36]. A pro-inflammatory and pro-oxidant milieu, especially in diabetes mellitus, stimulates PLT activation and ultimately causes occlusive thrombus formation; in turn, activation of coagulation induces multiple events leading to atherogenic inflammation and plaque complication [37].

In this framework, our data strongly suggest that SGLT2i, as a class, may exert a dual action, anti-inflammation in MAC and anti-aggregation in PLT, which may be plausible mechanism(s) to explain, at least partially, the early and significant reduction in the rates of CV outcomes in patients with established ASCVD, as demonstrated in several clinical trials [1, 2, 4].

### SGLT2i-mediated reduction of lipotoxicity in MAC

In our study, the anti-inflammatory effect of SGLT2i was accompanied by a decrease in SA-stimulated oxidant stress marker expression, supporting the hypothesis that lipotoxicity, via oxidant stress, might compromise MAC function, with consequent endothelial damage and plaque vulnerability.

In this study, gene expression and protein secretion data were relatively consistent. The divergent results—limited to IL-8 and MCP-1—might be reconciled by considering the substantial role played by the regulatory processes occurring after mRNA production (translation, mRNA and protein degradation/turnover) which have been acknowledged in the determination of discrepancy between transcriptomic and proteomic data [38, 39].

The parallel assessment of transcriptome and secretome revealed that MCP-1, the most important regulator of migration and infiltration of monocytes/macrophages, is indeed inhibited by SGLT2i—regardless of the lipotoxic stimulation—with an undoubted positive effect on atherosclerotic plaque stabilization. On the contrary, unlike gene expression data, the secretion of IL-8 seemed not to be affected by both empa and dapagliflozin, suggesting a fine regulation of protein stabilization—putatively required for vascular homeostasis maintenance-, due to IL-8 dual role in angiogenesis [40] and inflammation.

Anti-inflammatory and/or anti-oxidant effects of SGLT2i have been reported in animal models. SGLT2 inhibition with canagliflozin-treated ApoE^−/−^ mice showed a reduced inflammatory state—in terms of MCP-1 and VCAM expression—and increased plaque stability (improved metalloproteinase profile) compared to control mice [41]. Empagliflozin decreased myocardial oxidative stress markers and improved heart structure in a genetic type 2 mouse model by inhibiting TFGβ/Smad pathway [42]. On the other hand, a recent work has shown no effect of 12-weeks dapagliflozin treatment in pro-angiogenic cell bioavailability vs placebo in patients with T2D [43].

Taken together our results suggest that SGLT2i could positively affect MAC, by ameliorating their function rather than their bioavailability.

### SGLT2i-mediated reduction of PLT activation

PLT-mediated thrombus formation is a key step in vessel occlusion due to a complicated atherosclerotic plaque. PLT activation is triggered by subendothelial collagen—or by other agonist such as thrombin, thromboxane A2 and ADP—and is characterized by PLT morphological change and secretion of granule content (rich in ADP, serotonin, Ca^2+^, vWF and fibrinogen) [44]. Our study shows for the first time that SGLT2i blunt ADP-stimulated PLT activation, suggesting a role of these drugs in the attenuation/prevention of acute arterial occlusion following plaque rupture and consequent fatal or non-fatal CV events in SGLT2i treated patients. Importantly, these favorable effects were detected at the concentration of 1 µM corresponding to usual circulating levels in humans [45, 46].

### Possible molecular transducer(s) of EMPA and DAPA action in both cell populations

Another major study finding is that all the described effects due to MAC and PLT exposure to DAPA and EMPA were SGLT2 independent, as no SGLT2 gene or protein expression was detected in either cell population. SGLT1 expression was not assessed—although the recent signal of a possible expression in cardiomyocytes [47], due to the high selectivity of EMPA and DAPA for SGLT2 over SGLT1. Recent studies showed that SGLT2i can bind to NHE [8] and can improve cardiac function in rodent models through inhibition of myocardial NHE flux, thereby reducing cytoplasmic Ca^2+^ and Na^+^ concentrations. In line with these findings, we have found that inhibition of NHE results into a scenario of inflammation reduction in MAC and deactivation in PLT, reminiscent in both cases of the effects observed with cell exposure to DAPA or EMPA.

It has been long known that NHE blockade with amiloride can inhibit pro-inflammatory cytokine production and nuclear factor-kB activation in lipopolysaccharide (LPS)-stimulated endothelial cells [48] and macrophages [49, 50]. The present study extends this notion also to MAC, representing the first evidence of a potential connection between NHE inhibition and SGLT2i anti-inflammatory effects.

Accelerated Na^+^/H^+^ exchange plays an important role in the early phase of PLT [51]. Accordingly, amiloride [52] and cariporide (specific NHE1 inhibitor) [31] can dampen PLT activation. Importantly, amiloride, in comparison to β blockade, both in combination with hydrochlorothiazide, significantly reduced CV events in hypertensive subjects [53]. Finally, treatment with cariporide reduced all-cause mortality and myocardial infarction in patients undergoing coronary artery bypass [54].

Our results with cariporide provide clues about the contribution of the isoform NHE1 in SGLT2 inhibitor-mediated PLT de-activation and, at least partly, in EMPA anti-lipotoxic action. On the other hand, cariporide exhibited lesser effect than amiloride in curbing inflammation in MAC, thereby implying that more NHE isoforms are target of EMPA in MAC, possibly pointing to a role for NHE 6 which is differentially expressed in the two studied cell types. From our results it seems not possible to draw any definite conclusions on SGLT2i cell type effect on specific isoforms, beyond NHE1; further experiments are needed to clarify this issue.

Some study limitations need to be acknowledged: (1) lipotoxicity is a rather complex and multifactorial issue. In our study, we did not explore the effects of SGLT2 inhibition on PPARα and target genes expression which is known to regulate fatty acid metabolism and, ultimately, lipotoxicity [55, 56]. (2) In MAC experiments, EMPA and DAPA were effective at supra-pharmacological concentrations (100 µM), as compared to the circulating levels in humans (~ 1 µM); however, intra-cellular concentrations of SGLT2i under standard clinical conditions are unknown and concentrations between 10 and 100 µM of SGLT2i have been widely used in vitro [57, 58]; (3) although our data are compatible with a role of NHE inhibition in SGLT2i-mediated benefits in MAC and PLT, a causal relationship remains to be proven; (4) the extrapolation of our in vitro data to the complex in vivo setting should be made with caution.

## Conclusions

Our study shows that both EMPA and DAPA ameliorated lipotoxic damage in stearate-treated MAC, and reduced ADP-stimulated PLT activation, possibly via NHE-inhibition, thereby pointing to plaque stabilization and/or thrombosis inhibition as potential mechanism(s) involved in SGLT2i-mediated CV protection.

## Supplementary information

**Additional file 1: Figure S1.** Diagram of experimental procedures and results.

**Additional file 2: Figure S2.** NHE 7-9 isoform expressions from total protein extracts of PLT (on the left) and MAC (on the right) were assessed by western blotting. For eachdifferent antibody a positive control (cell extract of mouse liver or human lymphocytes) was used, as indicated in the datasheets.

## Data Availability

The datasets used and/or analyzed during the current study are available from the corresponding author on reasonable request

## Abbreviations

ASCVD: Atherosclerotic cardiovascular disease
CKD: Chronic kidney disease
CVOT: Cardiovascular outcome trials
DAPA: Dapagliflozin
EMPA: Empagliflozin
FFA: Free fatty acid
HO-1: Heme oxygenase
IL: Interleukin
MAC: Myeloid angiogenic cells
MACE: Major adverse cardiovascular events
MCP-1: Monocyte chemoattractant protein-1
MRF: Multiple risk factors
NAC: *N*-Acetyl-cysteine
NHEi: Sodium-proton exchanger inhibitor
PLT: Platelets
qPCR: Quantitative PCR
SA: Stearic acid
SGLT2: Sodium-glucose cotransporter 2
SGLT2i: Sodium-glucose cotransporter 2 inhibitors
SOD-2: Superoxide dismutase
T2D: Type 2 diabetes mellitus
TNF-α: Tumor necrosis factor-α
TXN: Thioredoxin
