# Supplementary figures and images for: Sodium-glucose cotransporter 2 inhibitors antagonize lipotoxicity in human myeloid angiogenic cells and ADP-dependent activation in human platelets: potential relevance to prevention of cardiovascular events
Source: Cardiovasc Diabetol. 2020 Apr 7;19:46. doi: 10.1186/s12933-020-01016-5 (PMC7140327; doi:10.1186/s12933-020-01016-5)

Figure S1

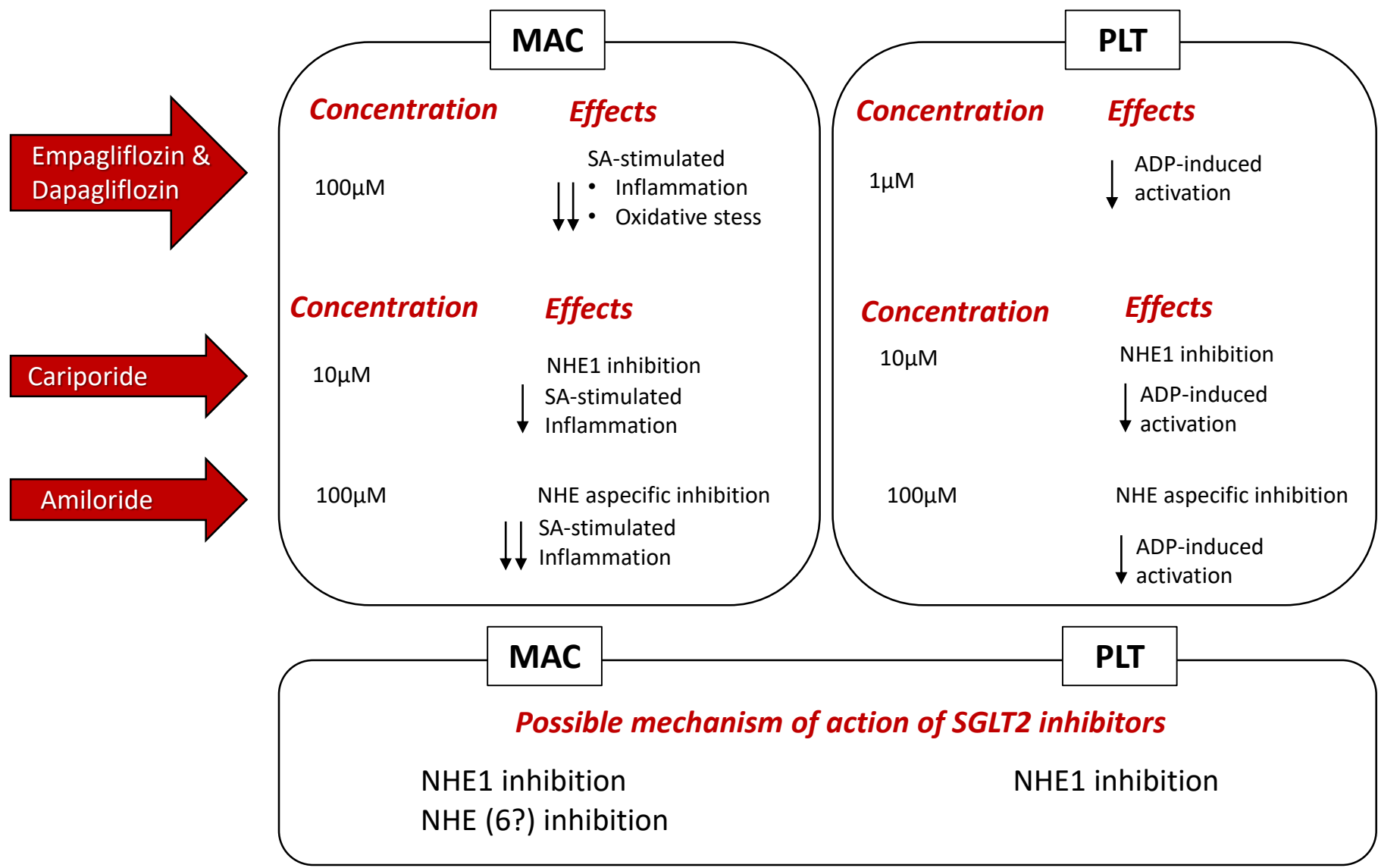

Supplement: Supplementary file 1 — Additional file 1: Figure S1. Diagram of experimental procedures and results. [file 12933_2020_1016_MOESM1_ESM.pdf]
