# Supplementary material for: Sodium-glucose cotransporter 2 inhibitors antagonize lipotoxicity in human myeloid angiogenic cells and ADP-dependent activation in human platelets: potential relevance to prevention of cardiovascular events
Source: Cardiovasc Diabetol. 2020 Apr 7;19:46. doi: 10.1186/s12933-020-01016-5 (PMC7140327; doi:10.1186/s12933-020-01016-5)
Supplement: Supplementary file 2 — Additional file 2: Figure S2. NHE 7-9 isoform expressions from total protein extracts of PLT (on the left) and MAC (on the right) were assessed by western blotting. For eachdifferent antibody a positive control (cell extract of mouse liver or human lymphocytes) was used, as indicated in the datasheets. [file 12933_2020_1016_MOESM2_ESM.pdf]

Figure S2

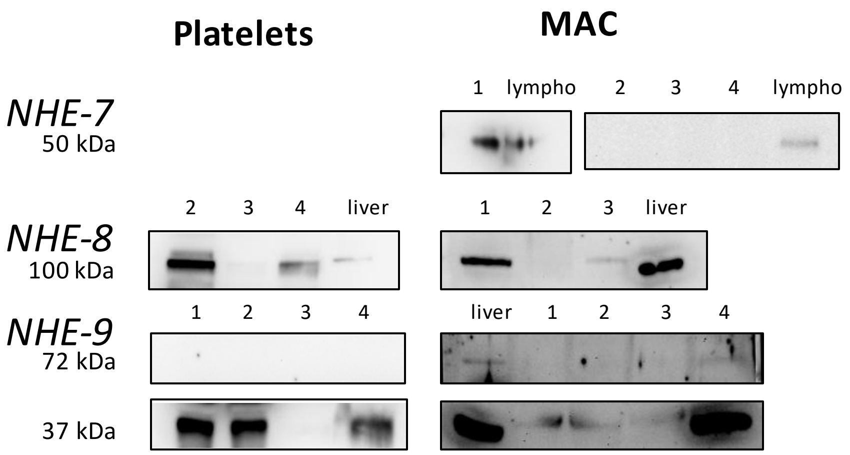

Figure S2. NHE 7-9 isoform expressions from total protein extracts of PLT (on the left) and MAC (on the right) were assessed by western blotting. For each different antibody a positive control (cell extract of mouse liver or human lymphocytes) was used, as indicated in the datasheets.
